# Anti-Penetration Performance of Composite Structures with Metal-Packaged Ceramic Interlayer and UHMWPE Laminate

**DOI:** 10.3390/ma16062469

**Published:** 2023-03-20

**Authors:** Xin Sun, Longhui Zhang, Qitian Sun, Ping Ye, Wei Hao, Peizhuo Shi, Yongxiang Dong

**Affiliations:** 1 State Key Laboratory of Explosion Science and Technology, Beijing Institute of Technology, Beijing 100081, China; 2 Explosion Protection and Emergency Disposal Technology Engineering Research Center of the Ministry of Education, Beijing Institute of Technology, Beijing 100081, China

**Keywords:** metal-packaged ceramic interlayer, UHMWPE laminate, anti-penetration performance, failure mechanisms

## Abstract

The impact response of a composite structure consisting of a metal-packaged ceramic interlayer and an ultra-high molecular weight polyethylene (UHMWPE) laminate has been studied through a ballistic test and numerical simulation. The studied structure exhibits 50% higher anti-penetration performance than the traditional ceramic/metal structure with the same areal density. The metal-packaged ceramic interlayer and the UHMWPE laminate are key components in resisting the penetration. By using a metal frame to impose three-dimensional constraints on ceramic tiles, the metal-packaged ceramic interlayer can limit the crushing of the ceramic and contain the broken ceramic fragment to improve the erosion of the projectile. The large deformation of UHMWPE laminate absorbs a large amount of energy from the projectile. By decreasing the amplitude of the shock wave and changing the distribution of the impact load in the structure, the projectile has longer residence time on the interlayer. The anti-penetration performance shows within 10% variation when the impact position is varied. Due to the asymmetric deformation and high elastic recovery ability of the UHMWPE laminate, the projectile trajectory deflection is increased, and the broken ceramic fragments are restrained, thereby mitigating after-effect damage caused by the projectile after penetrating the structure.

## 1. Introduction

Ultrahigh molecular weight polyethylene (UHMWPE) fiber is increasingly used in protective structures because it has extremely high specific tensile strength and stiffness [1,2]. With UHMWPE, composite structures can have much lower areal density and much better anti-penetration performance [3]. Extensive research on the deformation and failure mechanisms of UHMWPE [4,5,6] reveals that thanks to its high elastic modulus, the UHMWPE fiber can quickly disperse strain waves and rapidly absorb the kinetic energy from an impact [7,8,9,10]. Attwood et al. [11] numerically and experimentally examined the compressive response of quasi-static out-of-plane UHMWPE laminate and found that the indirect tension mode of deformation and failure occurs because of anisotropy.

When impacted by a projectile, the UHMWPE laminate can undergo two stages of plastic deformation [12]. The first is the bulge deformation stage, during which the impact stress propagates in the transverse direction, the plastic deformation zone of the fiber expands, and the height and edge width of the laminate protrusion increase with impact time [13]. The second is the limited deformation stage. The UHMWPE laminates can resist penetration by a projectile with a sharp head because the fibers at the point of impact are sheared, and the surrounding fibers are stretched [14]. In addition, the laminate can be deformed to a greater degree through layering, and the energy of the projectile can be fully offset by stretching the fibers and films [15]. By comparing the anti-penetration performance of UHMWPE laminates of three different woven structures, it was found that the unidirectional prepreg (UD) construction had the best performance [16].

Nevertheless, a protective structure using UHMWPE fiber alone has a small ballistic limit because the UHMWPE laminate has smaller compressive strength than metal projectiles. Instead, UHMWPE laminate is usually combined with metal and ceramic materials to resist projectile penetration. The UHMWPE laminate exhibits good anti-penetration performance under impact loads in composite structures consisting of Ti6Al4V (TC4) plate, cylindrical ceramics, and UHMWPE laminate [17]. In view of the high strength and toughness of TC4, the composite laminate of TC4 and UHMWPE can be ideal as a backing material [18].

Traditional composite protective structures mostly make use of ceramic and metal materials [19,20,21,22,23] and mostly adopt a layered structure (monolithic ceramic as interlayer) or a two-dimensional frame-restraint structure (strip-shaped ceramics as interlayer) [24,25,26,27,28]. The traditional ceramic/metal composite structure tends to have stringent requirements on material performance, which renders poor flexibility in design [29]. Most of the existing studies focus on the layered structure of the whole ceramic, but the research on the anti-penetration performance of the ceramic composite structure with three-dimensional metal packaging is less.

Currently, there is a rising demand for protective structures that has lower areal density and higher anti-penetration performance, but the traditional composite structures cannot meet these demands [30,31,32]. To overcome the drawbacks of the monolithic ceramic interlayer in composite protective structures, we used a ballistic test and numerical simulation to investigate the anti-penetration performance and failure mechanisms of a composite structure that comprises a metal-packaged ceramic interlayer and UHMWPE laminate, aiming to provide scientific guides for the design of high-performance composite structures.

## 2. Target Description

**Figure 1:**
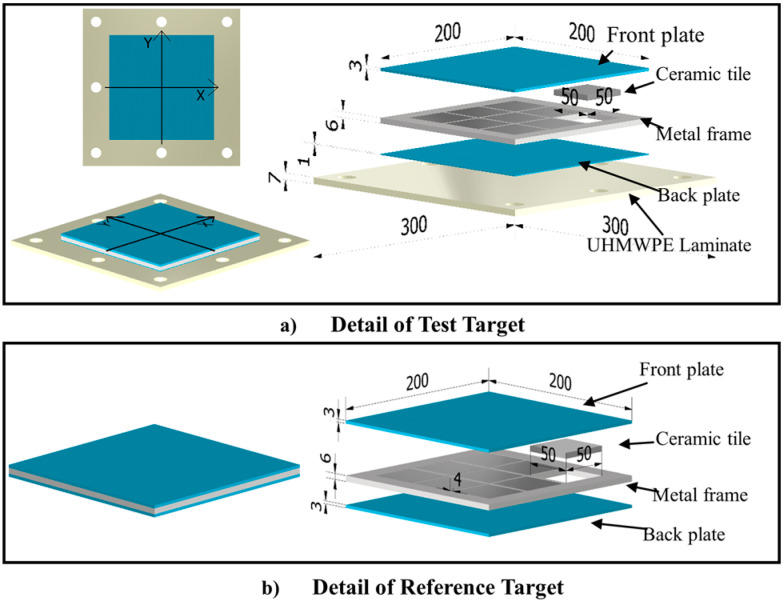
Schematic diagram of the test and reference targets. (**a**) Detail of Test Target. (**b**) Detail of Reference Target.

The composite protective structure comprises a front plate, an interlayer with 3 × 3 ceramic tiles packed in a metal frame, a back plate, and a UHMWPE laminate (Figure 1a). Table 1 lists the dimensions of the individual parts, which are bound together by two-component epoxy adhesive. The dimensions of the metal panel, the metal-packaged ceramic interlayer, and the metal backplane are 200 × 200 mm; the dimension of the UHMWPE laminate is 300 × 300 mm; the metal-packaged ceramic interlayer is formed by combining 50 × 50 mm SiC ceramic tiles and a metal frame whose width between two ceramic tiles is 4 mm.

The UHMWPE laminate (0.97 g/cm^3^) is made of UHMWPE fiber (41 g/d) through two-dimensional orthogonal weaving. Holes are punched around the UHMWPE laminate to fix it to the holder with bolts because it can be easily pulled inward from the boundary by the restricting clamps during the penetration [14]. All metal parts, including the front plate, the back plate, and the metal frame in the interlayer, are made of Ti-6Al-4V (TC4) and fabricated by wire cutting. The composite structure has a low areal density of 45 kg/m^2^. A traditional ceramic/metal composite structure with the same areal density serves as the reference target (Figure 1b).

## 3. Experiment Analysis

### 3.1. Ballistic Test

**Figure 2:**
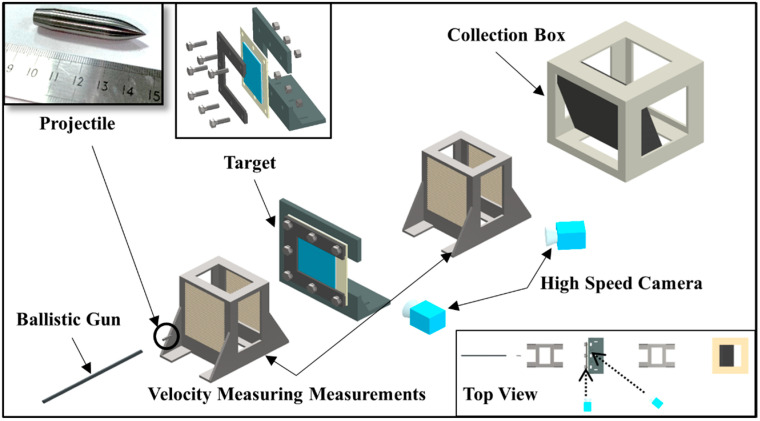
Schematic diagram of the ballistic testing experimental layout.

The experimental setup of the ballistic test comprises a ballistic gun, a U-shaped steel holder, four velocity measuring measurements, two 710 high-speed cameras (Vision Research, Inc., Wayne, NJ, USA), and a recovery bin filled with rubber plates (Figure 2). The projectile is a sharp-nosed bullet (30 g, *Φ* = 10.8 mm) fired from a ballistic gun that penetrates the target perpendicularly, and it is loaded with a nylon sabot to ensure the stability of its movement in the barrel. The four velocity measuring measurements (in two groups, at the front and back of the target) measure the initial and residual velocity of the projectile. The high-speed camera at the side captures the flight, penetration, and after-effect of the projectile. The high-speed camera at the back captures the deformation process of the target during the penetration of the projectile. The U-shaped steel holder is used to fix the target. The UHMWPE laminate and the holder are connected by bolts to reduce the pull-in of the UHMWPE laminate during the penetration. The collection box filled with rubber plates is used to retrieve the projectile after the penetration.

**Figure 3:**
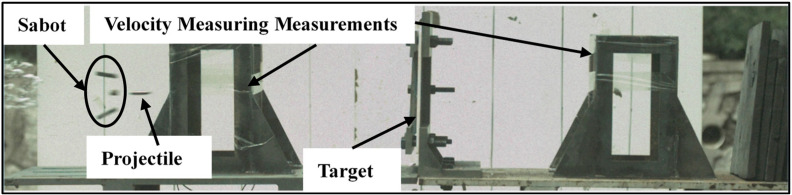
The high-speed camera images of the projectile impacting the target.

Figure 3 shows on the high-speed camera images that the projectile hits and penetrates the target only after the sabot is fully detached, and it can be considered that the readings of the velocity measuring measurements can accurately reflect the impact velocity of the projectile. Through high-speed camera images, the angle of each impact in five ballistic experiments was found being very close to 90°. Therefore, it can be considered that all impacts are vertical.

### 3.2. Experimental Results and Discussion

Table 2 lists the experimental results of five ballistic experiments, including projectile impact velocity, residual velocity, and projectile residual mass. Whereas the reference target is penetrated by the incoming bullet at 608 m/s, the test target successfully prevents penetration when the incoming projectile has a velocity of 592 m/s. In other words, adding the UHMWPE laminate to the composite structure improves the anti-penetration performance of the target. As shown in Table 2, under the same impact velocity, the projectile does not penetrate the test target when it hits the center position of the test target plate, such as T-X, but when the impact point is off-center, such as T-O, the projectile will penetrate the test target. Therefore, when the impact load is held unchanged, the anti-penetration performance of the test target appears to depend on the positions of impact. However, this situation is not obvious in the reference target board, comparing R-1 and R-2.

Table 3 shows the deformation of each structural component of the target after the impact. For all targets, the perforation has a larger diameter on the back plate than on the front plate, which means the impact load of the projectile experiences considerable lateral transmission in the interlayer. As a result, the impact load area expands on the back plate, and the anti-penetration performance of the composite structure is improved. The UHMWPE laminate absorbs the energy of the projectile by undergoing significant deformation in all three dimensions.

**Figure 4:**
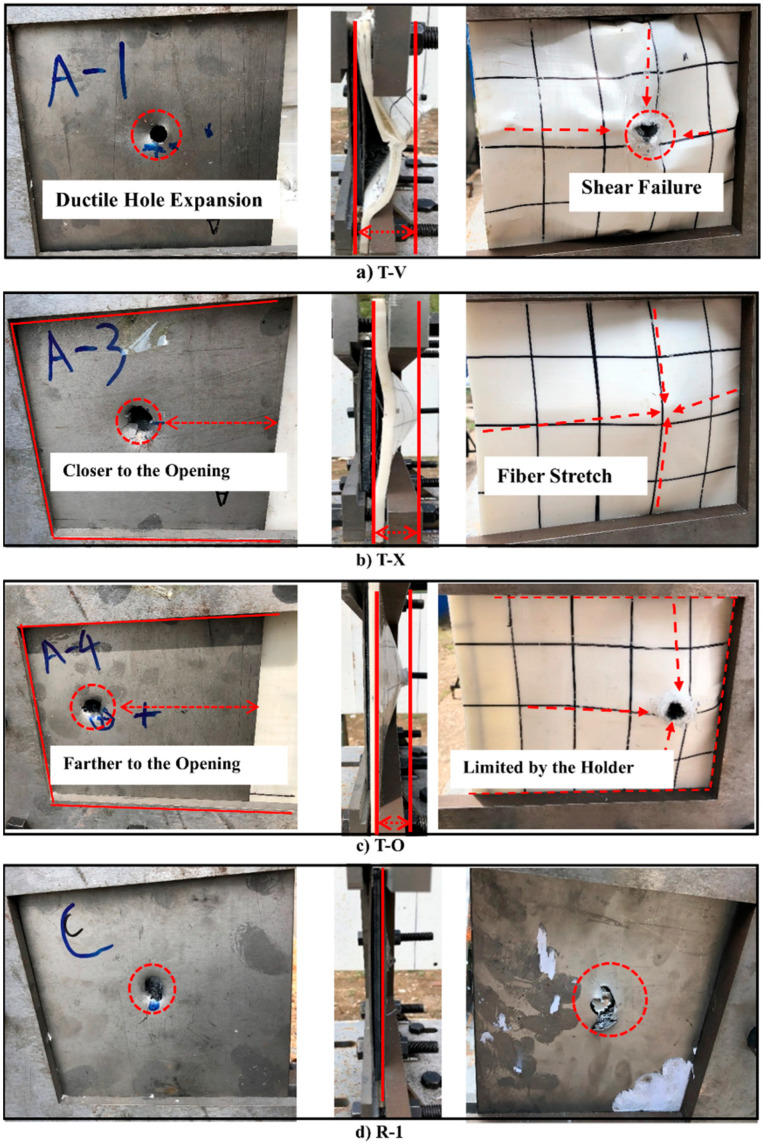
Pictures of the test and reference target after projectile impact. (**a**) Impact test of T-V. (**b**) Impact test of T-X. (**c**) Impact test of T-O. (**d**) Impact test of R-1.

Figure 4 shows the deformation and damage of the test target and reference target after the impact. Both the front plate and the back plate are damaged at the point of impact, on both of which an expanded ductile hole is formed due to the thinness of the metal plate as well as the high velocity and the sharp head of the projectile. Compared with the metal plates, the UHMWPE laminate deforms to a greater extent after the impact, but its degree of deformation is limited by the steel holder.

## 4. Numerical Analysis

### 4.1. Numerical Simulation Model

**Figure 5:**
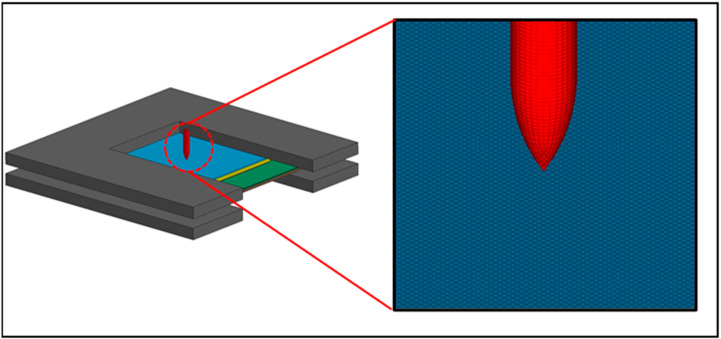
Finite element model of the composite structure.

Figure 5 shows the 3D finite element model built with the LS-DYNA software (R11.2.0, Livermore Software Technology, LLC, Livermore, CA, USA). The composite structure is divided by regular hexahedral grids (0.5 × 0.5 mm). The projectile uses gradient grids, with the innermost grid also being 0.5 × 0.5 mm [33], and other grids generated automatically in the radial direction. To ensure simulation accuracy, the longest side of the gradient grid is kept within 0.8 mm in length. In the experiment, the UHMWPE laminate consists of 48 orthogonal prepreg sheets, each with a thickness of about 0.15 mm. In this model, due to the limitation of computational capacity, three prepreg fabrics are combined into one layer with a thickness of 0.46 mm [14]. Because the size of the UHMWPE laminate was large enough in the experiment, the material at the bolt hole was almost not deformed, so the boundary of the bolt hole was not modeled.

The Lagrangian algorithm is adopted for the projectile and composite structure. The element type is a hexahedral solid element with reduced integral. In order to avoid the hourglass deformation, the HOURGLASS and CONTROL_HOURGLASS keywords are used for control, and the hourglass control type is the Flanagan–Belytschko stiffness form. The CONTACT_ERODING_SURFACE_TO_SURFACE keyword is used for the projectile in penetration. The CONTACT_AUTOMATIC_SURFACE_TO_SURFACE keyword is used for the automatic contact that considers the characteristics of large impact deformation. The CONTACT_TIEBREAK_SURFACE_TO_SURFACE keyword is set between the corresponding parts to better simulate the adhesion between the ceramic tiles and the metal frame. In this keyword, the NFLS (Tensile failure stress) value is 120 Mpa, and SFLS (Shear failure stress) value is 60 Mpa. The connections among the UHMWPE laminates in detail use CONTACT_TIEBREAK_SURFACE_TO_SURFACE to simulate. In this keyword, NFLS value is 950 Mpa, and SFLS value is 950 Mpa.

The Johnson–Cook material model is used for the metal material to simulate the dynamic response characteristics of the target action (Table 4). The MAT_ADD_EROSION keyword was used to control the compression failure, and the value of MXEPS was set to 0.5. The Johnson–Holmquist-2 ceramics material model is selected for the ceramic material (Table 5) [34]. In this keyword, the FS value is 1.3.

The macroscopic representation of UHMWPE laminates is orthotropic. For this orthotropic material, its constitutive relation can be expressed as:

(1) $$\left\{\begin{array}{c} \varepsilon_{1}\\ \varepsilon_{2}\\ \begin{array}{c} \begin{array}{c} \varepsilon_{3}\\ \gamma_{23} \end{array}\\ \gamma_{31}\\ \gamma_{12} \end{array} \end{array}\right\}=\left\{\begin{array}{cc} \begin{array}{ccc} \frac{1}{E_{1}} & \frac{{-\nu }_{12}}{E_{1}} & \frac{{-\nu }_{13}}{E_{1}}\\ \frac{{-\nu }_{12}}{E_{2}} & \frac{1}{E_{2}} & \frac{{-\nu }_{23}}{E_{2}}\\ \frac{{-\nu }_{13}}{E_{3}} & \frac{{-\nu }_{23}}{E_{3}} & \frac{1}{E_{3}} \end{array} & 0\\ 0 & \begin{array}{ccc} \frac{1}{G_{23}} & 0 & 0\\ 0 & \frac{1}{G_{13}} & 0\\ 0 & 0 & \frac{1}{G_{12}} \end{array} \end{array}\right\}\left\{\begin{array}{c} \begin{array}{c} \sigma_{1}\\ \sigma_{2}\\ \sigma_{3} \end{array}\\ \begin{array}{c} \tau_{23}\\ \tau_{13}\\ \tau_{12} \end{array} \end{array}\right\}$$

where *E*, *G* and ν were the elastic modulus, shear modulus and Poisson ratio of the UHMWPE laminate, and the subscripts 1, 2, and 3 denoted local element axes. Based on the test results of dynamic mechanical properties of UHMWPE fiber and its laminates [35,36,37], the material model parameters of UHMWPE laminates were obtained by meso-mechanical theory calculation. The UHMWPE laminate material adopts the composite damage material model (Table 6).

### 4.2. Model Verification

**Figure 6:**
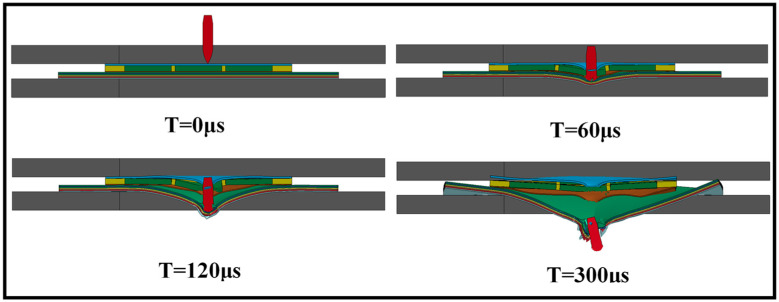
Simulation of the penetration of T-V by the projectile at 673 m/s.

**Figure 7:**
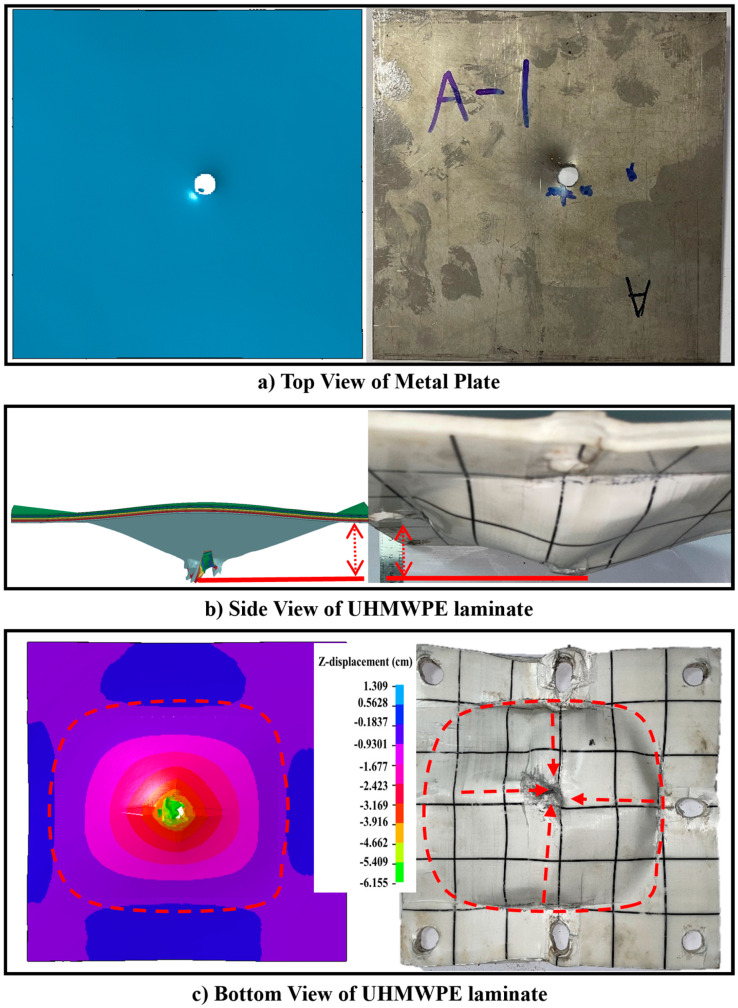
Simulated damage and deformation of front plate and UHMWPE laminate. (**a**) Top view. (**b**) Side view. (**c**) Bottom view.

**Figure 8:**
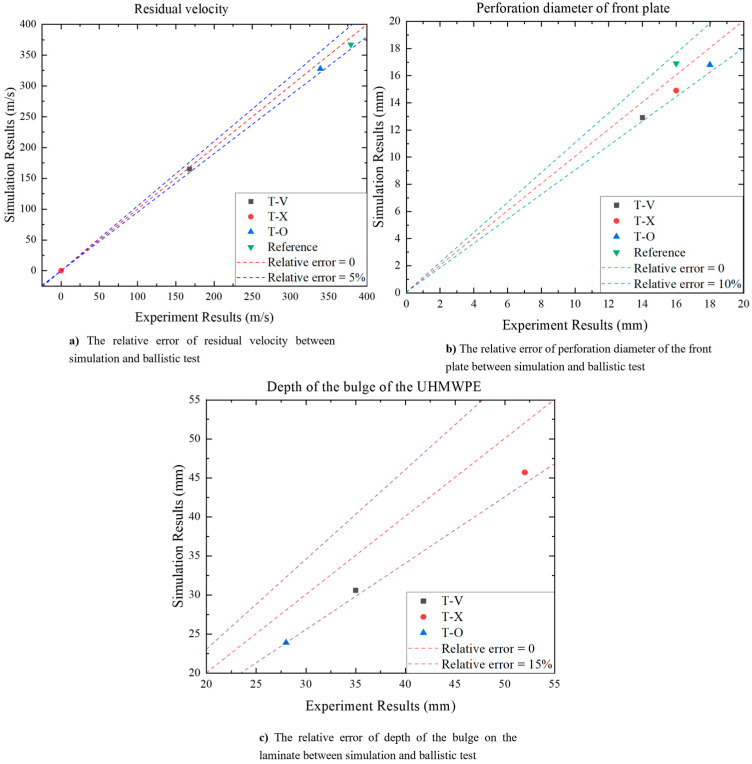
The relative error of simulation and ballistic tests. (**a**) Residual velocity. (**b**) Perforation diameter of the front plate. (**c**) Depth of the bulge on the laminate.

The process of a projectile impacting a T-V target plate at 673 m/s speed is shown in Figure 6. It can be seen that the projectile finally penetrates the target plate and deflects the ballistic trajectory. For T-V, the front plate and the UHMWPE laminate basically have the same failure form and degree of deformation (Figure 7). Table 7 compares the results from the simulation and the ballistic test, including the residual velocity of the projectile, the perforation diameter of the front plate, and the deformation of the UHMWPE laminate. The comparison shows that the simulated residual velocity of the projectile in the four ballistic tests compared in Table 7 is in good agreement with the experimental results, and the maximum error is only 3%, as shown in Figure 8a. For the perforation diameter of the front plate and the depth of the bulge on the laminate, the error is less than 13%, as shown in Figure 8b,c. Therefore, the simulation results are in good agreement with the test results.

### 4.3. Numerical Results and Discussion

#### 4.3.1. The Anti-Penetration Performance of Studied Target

**Figure 9:**
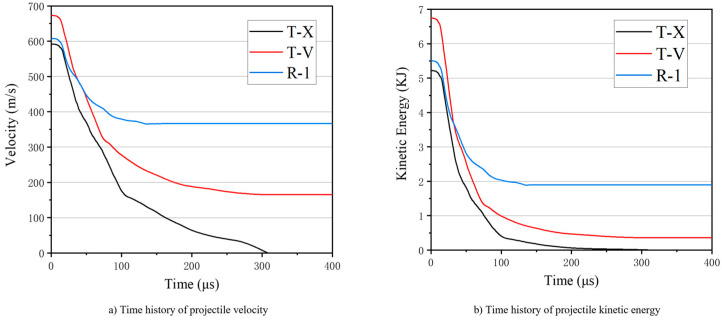
Time history of projectile velocity (**a**) and kinetic energy (**b**) during the penetration.

Figure 9a,b plots the simulated velocity and kinetic energy of the projectile against time to analyze the penetration process. It can be seen from the results of T-X and R-1 that the anti-penetration performance of the target is improved by up to 50% after the UHMWPE laminate is added to the bare ceramic/metal composite structure. For the test targets, the projectile decelerates rapidly after 10–70 μs of impact, during which time it collides with the metal-packaged ceramic interlayer (Figure 6).

#### 4.3.2. The Failure Mechanisms of Studied Structure during the Penetration

**Figure 10:**
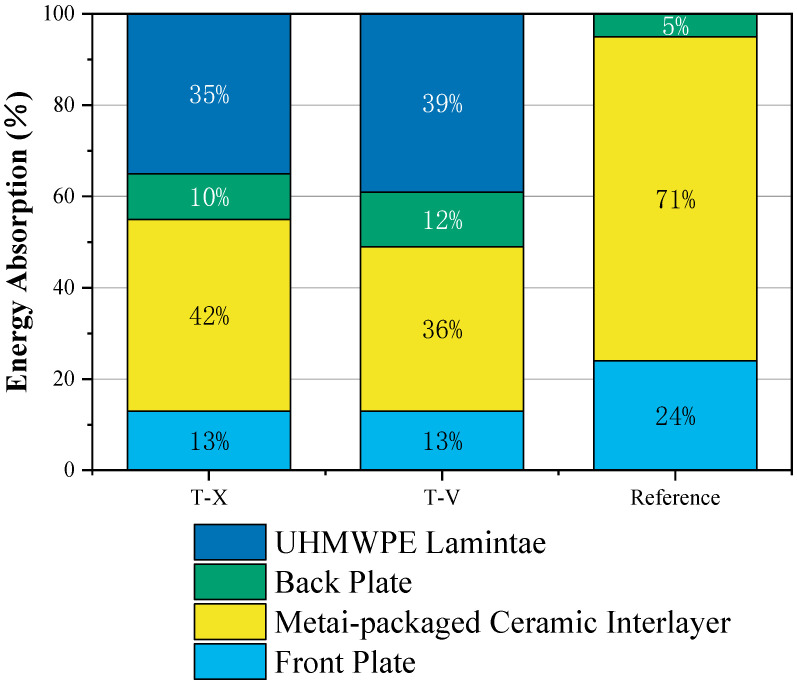
Breakdown of energy absorption within the composite structure.

The most important energy-absorbing component for the test target is the metal-packaged ceramic interlayer and the UHMWPE laminate. Figure 10 presents the energy absorption ratio of each part of the composite structure for the target in the anti-penetration process. The energy absorption of each part is obtained through the change of the internal energy and kinetic energy of each part during the penetration process and the change of the erosion energy of the projectile when penetrating each part.

The interlayer clearly absorbs a large amount of energy and rapidly reduces the projectile’s velocity at the beginning of the impact. Ceramic is a brittle material that can be easily broken to absorb the energy of the penetrating projectile. Because of its high rigidity and large compressive capacity, ceramic readily erodes incoming projectiles and reduces their mass. The projectile starts to deform once its head reaches the ceramic interlayer and constant erosion then starts (Figure 6). However, ceramic has poor tensile strength, and the cracks generated as the ceramic is crushed are rapidly propagated, which decreases the strength of the ceramic tiles. To limit the crushing of the ceramic and contain the broken ceramic fragments, a metal frame can be used to divide the whole interlayer into 3 × 3 ceramic tiles and impose three-dimensional constraints.

**Figure 11:**
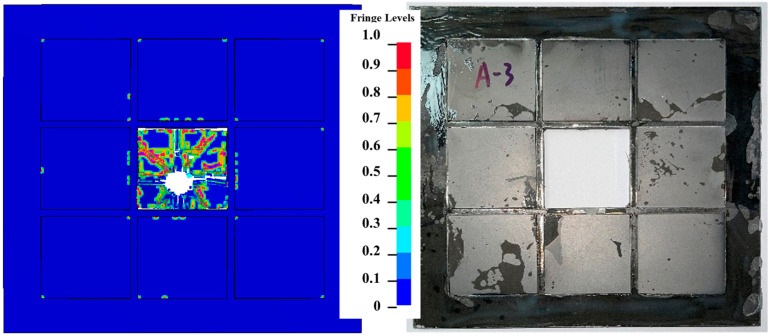
Damage contour of the ceramic tiles in T-X.

Figure 11 shows the destruction of ceramic materials after the penetration of the T-X. Only a small area of the ceramic is damaged during the penetration process because the metal frame in the interlayer limits the expansion of cracks.

**Figure 12:**
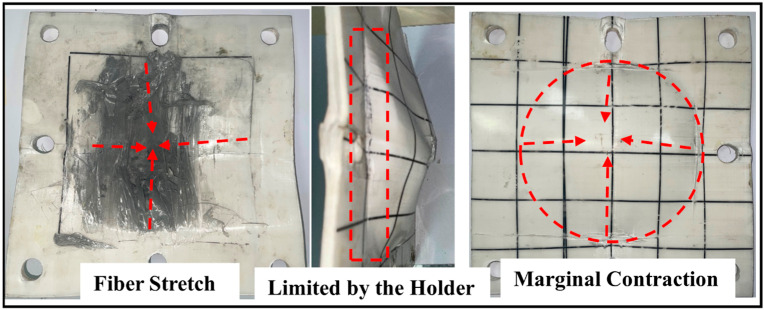
Marginal contraction of the UHMWPE laminate during the bulging stage.

Figure 12 shows that the UHMWPE laminate in the T-X target, due to its strong tensile strength, is not penetrated but forms an inverted cone-shaped bulge. Because the fibers of the UHMWPE laminate are arranged orthogonally, at the time of impact, the fibers further away from the point of impact are stretched and then move inward. Therefore, under the impact load, the UHMWPE laminate can deform to absorb a large amount of energy from the projectile before its fibers are sheared.

**Figure 13:**
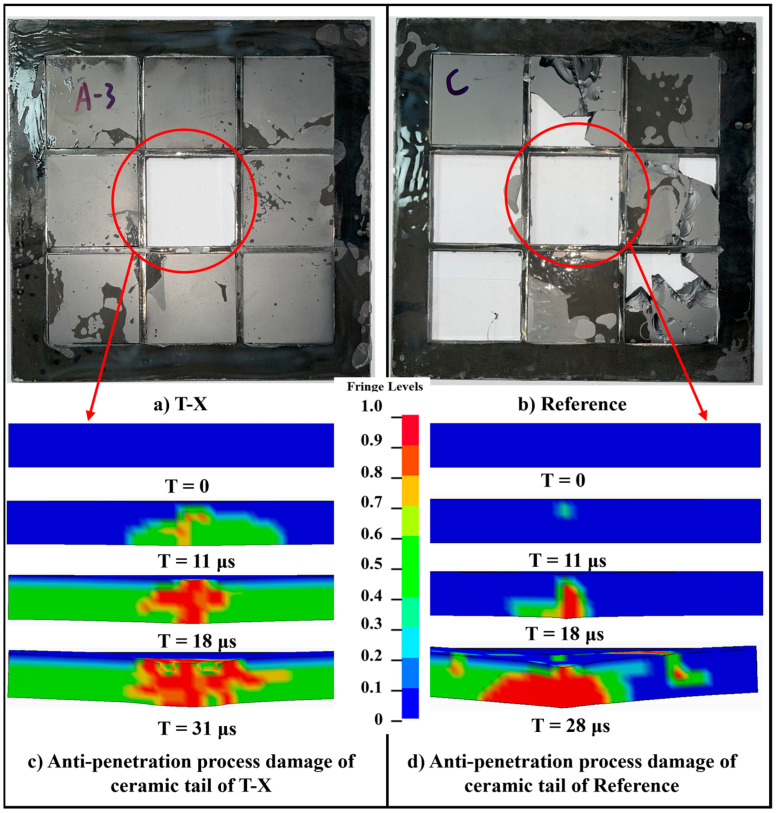
The anti-penetration process damage of ceramic tail of different targets. (**a**) Experiment result of ceramic tiles damage of T-X. (**b**) Experiment result of ceramic tiles damage of Reference. (**c**) Simulation result of ceramic tiles damage of T-X. (**d**) Simulation result of ceramic tiles damage of Reference.

The test target with a composite back plate of metal sheet and UHMWPE laminate has a later damage time of ceramic penetration during the anti-penetration process than that of the reference target with only a metal back plate, as shown in Figure 13. At the same time, as shown in Figure 13a,b, the final damage influence range of the ceramic tiles T-X is much smaller than that of R-1, where almost all ceramic tiles in the reference target are damaged, while for T-X, only the ceramic tile at the impact point of the projectile is damaged, thus making it still capable of resisting the next impact. This is mainly because the UHMWPE laminate changes the load distribution and reduces the stress peak of the ceramic tiles during the penetration process through its better deformation properties.

**Figure 14:**
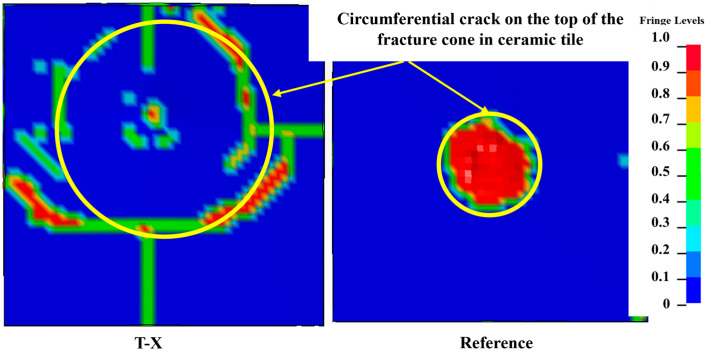
The circumferential crack on the top of the ceramic of different targets.

The load distribution of the ceramic tiles during the anti-penetration process changes due to the UHMWPE laminate, which results in a wider range of circumferential cracks on the top of the fracture cone than the reference target without UHMWPE laminate, as shown in Figure 14. Larger circumferential cracks can not only increase the absorbed projectile energy by expanding the crushing range of the ceramic tiles but also prolong the residence time of the projectile on the ceramic surface [39], which explains that the ceramic tiles in the T-X developed penetrating damage later, as shown in Figure 13.

**Figure 15:**
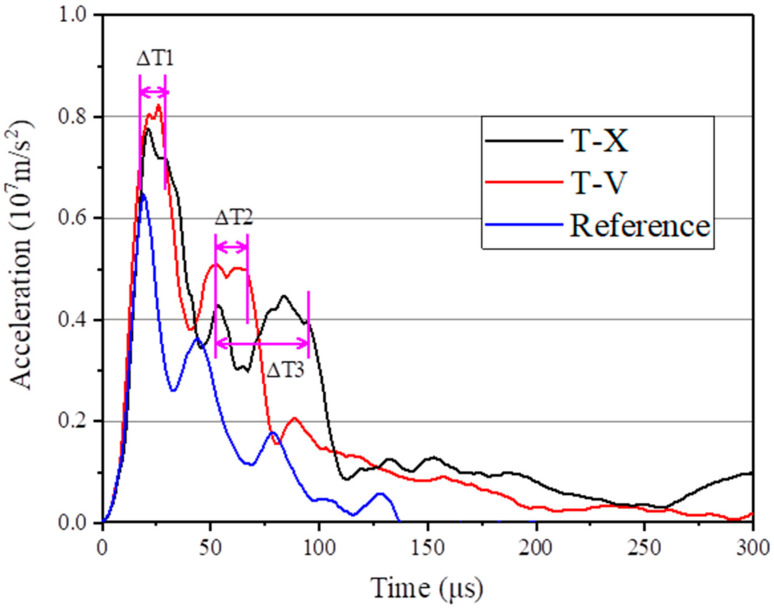
Time history of projectile acceleration during the penetration.

In order to quantify the residence time of the projectile on the ceramic surface during the anti-penetration process of different targets, the time–history curve shown in Figure 15 was obtained by extracting the overload curve of the projectile during the anti-penetration process. The acceleration of the projectile has two plateau periods during the penetration of T-X and T-V, but no such plateau exists for the penetration of R-1. That is, for the reference target, the ceramic material quickly breaks down during the penetration of the projectile. For the test target equipped with UHMWPE laminate, the projectile remains in the ceramic interlayer, similar to the projectile residence phenomenon.

First, the UHMWPE laminate provides strong support to the back plate by absorbing the energy of the projectile through deformation. Second, the UHMWPE laminate changes the distribution of the impact load in the composite structure. As a result, the projectile has longer residence time in the interlayer because it reaches the tensile strength of the ceramic tile at a later time than in the reference target.

The first platform of deceleration occurs at an early stage when the projectile freshly comes into contact with the target. At this time, the back plate is not yet deformed, the ceramic tiles are not damaged, and the target exerts a relatively large counterforce to the projectile. The second platform lasted longer, but the acceleration value was lower, which occurred when the shock wave generated by the project impact deformed the UHMWPE laminate. Upon such deformation, the support of UHMWPE laminate to the back plate is reduced, causing the ceramic tiles to crack and lose strength. As a result, the projectile begins to penetrate the ceramic tiles, although it still experiences strong counterforce during this time because the ceramic tiles are not completely broken. In the composite structure, the front plate limits the scattering of broken ceramics, and the back plate provides sufficient support to the ceramic interlayer.

**Figure 16:**
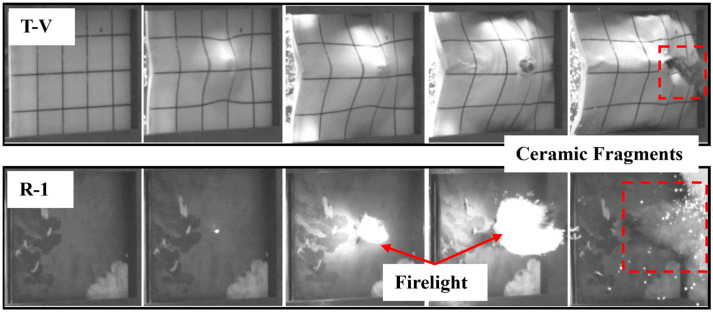
High-speed image sequences of T-V and R-1.

**Figure 17:**
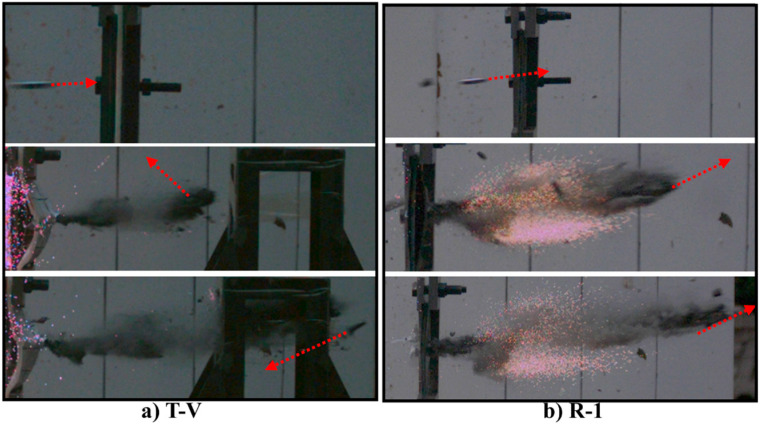
High-speed images of the change of warhead direction during penetration. (**a**) Impact test of T-V. (**b**) Impact test of R-1.

Figure 16 shows the high-speed image sequences for the penetration of R-1 and T-V, both of which are penetrated but experience different after-effects with regard to their damage response after the penetration. Compared with the reference target, the test target has fewer scattered ceramic fragments and no firelight after the penetration. The mitigated after-effect of the test target may be attributed to the high elastic recovery ability of the UHMWPE laminate, which, after the penetration, can retract the fibers to seal the perforation, thereby preventing the fire and ceramic fragments from flying further. In addition, compared with the reference target, the test target deflects the trajectory of the projectile significantly with a deflection angle of >180° (Figure 17), which can be mainly attributed to the asymmetrical deformation of the UHMWPE laminate. Specifically, for the test targets, the projectile is subject to an asymmetrical force from the UHMWPE laminate that reinforces the ballistic deflection.

#### 4.3.3. The Anti-Penetration Performance at Different Impact Positions

The projectile’s point of impact on the target also affects the anti-penetration performance of the target because the steel holder restricts the deformation of the UHMWPE laminate asymmetrically. Figure 4 shows that the projectiles essentially hit T-X, T-V, and R-1 at the geometric center but hit T-O farther from the opening of the steel holder. Figure 4c clearly shows how the steel holder restricts the deformation of the UHMWPE laminate, as a result of which a linear deformation area is formed at the edge of the circular deformation area. Although in the test T-O and test T-X, the test targets receive the same impact load, it behaves with worse resistance to penetration in test T-O than in test T-X mainly because, for T-O, the point of impact is away from the geometric center of the target and the deformation range of the UHMWPE laminate is limited by the steel holder.

**Figure 18:**
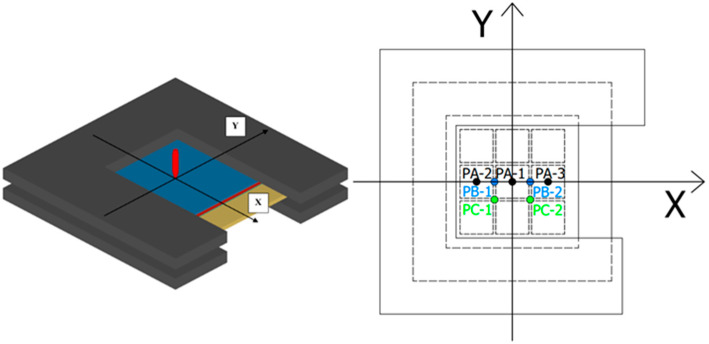
Finite element models of different impact positions.

The different outcomes of T-X and T-O indicate that the impact position of the projectile affects the anti-penetration performance of the composite structure. Although the velocity of the projectile is the same for both targets, the projectile cannot breach T-X but penetrates T-O with a residual velocity of 339 m/s. In addition, compared with T-X, T-O has both a smaller deformation range and a shallower bulge on the UHMWPE laminate (Table 3). Thus, the penetration of targets impacted at varying positions is studied further in the numerical simulation (Figure 18).

**Figure 19:**
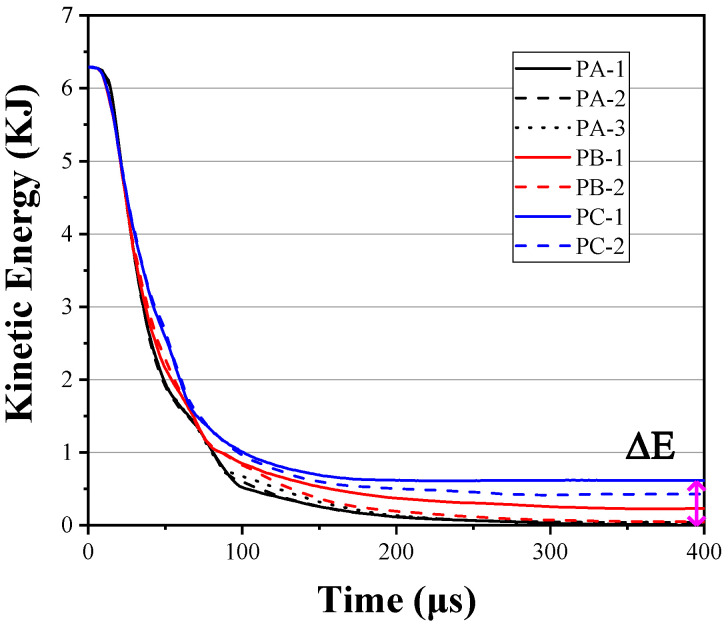
Time history of projectile kinetic energy at varying impact positions.

Table 8 shows that the projectile cannot penetrate when it hits the center of the target at 650 m/s (PA-1), and the resistance to penetration is stronger when the point of impact is closer to the opening of the steel holder as the impact position moves along the line of symmetry of the steel holder (PA-3 and PA-2). This is the opening side of the U-shaped steel holder and does not restrict the deformation of the UHMWPE laminate as much as the other sides. Consequently, when the point of impact is closer to the opening side (as in PA-3), the UHMWPE laminate has a larger area of deformation and a deeper bulge. Figure 19 shows the kinetic energy history of projectiles at varying the impact position. The difference in the anti-penetration performance of test targets is less than 10% when the impact position is varied.

**Figure 20:**
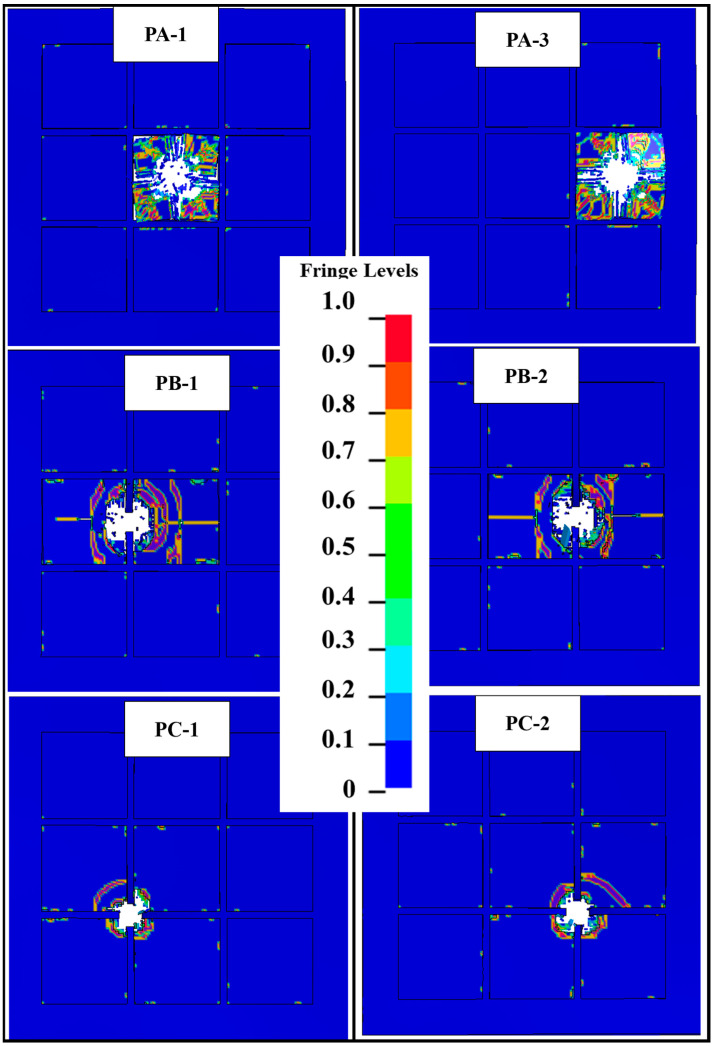
Damage of ceramic material of PA, PB, and PC after impact.

Figure 20 shows that the degree of ceramic fragmentation is different when the impact position has deviated systematically away from the center of the ceramic tiles. Table 8 shows that the simulated targets PA-2, PB-1, and PC-2 have largely the same bulge depth on UHMWPE laminate, although their anti-penetration performance differs notably. Presumably, the impact position changes the degree of ceramic fragmentation in the metal-packaged ceramic composite interlayer and varies the energy absorption. In addition, it can be seen by comparing PB-1 with PB-2 and PC-1 with PC-2 that when the degree of ceramic fracture is the same, the main factor that affects the anti-penetration performance of the composite structure is the degree of deformation of the UHMWPE laminate.

**Figure 21:**
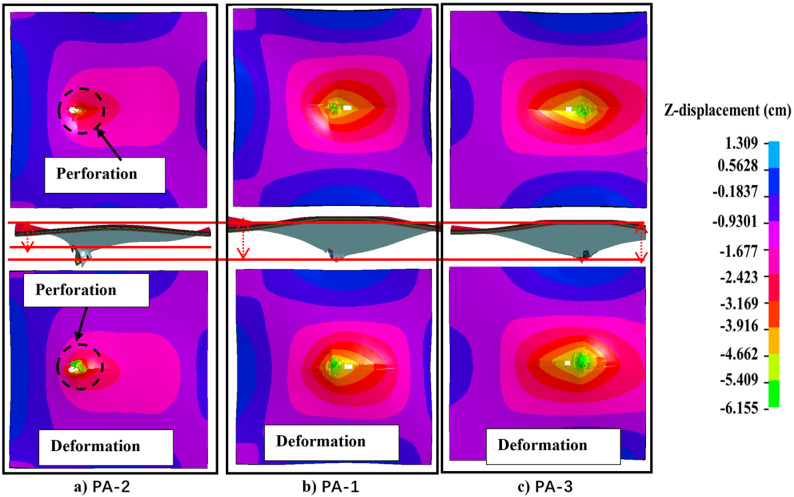
Deformation of UHMWPE laminate during penetration of PA at different hit points PA-2 (**a**), PA-1 (**b**) and PA-3 (**c**).

The deformation of the UHMWPE laminate on the back of the protective structure when the projectile hits different points is shown in Figure 21. The deformation range and ridge height of the UHMWPE laminate in PA-2 are smaller than those of PA-1 and PA-3. The deformation range of the UHMWPE laminate in PA-2 is larger than that in PA-1. This is mainly because the asymmetric target frame constraints make the deformation of the UHMWPE laminate in the positive and negative directions of the *Y* axis and the negative direction of the *X* axis constrained. Therefore, these reasons lead to the range of deformation asymmetry.

**Figure 22:**
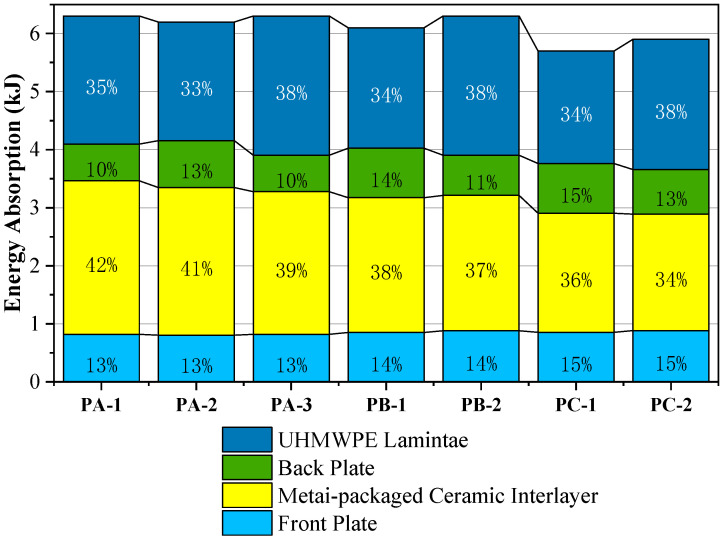
Breakdown of energy absorption within the composite structure at varying impact positions.

The energy absorption of each part of the protective structure during the projectile penetrating different positions is shown in Figure 22. About 5% and 8% of the difference in the anti-penetration performance comes from the degree of ceramic fragmentation and the degree of deformation of the UHMWPE laminate, respectively.

## 5. Conclusions

The current work adopted a ballistic test and numerical simulation to assess the anti-penetration performance and mechanism of a composite structure that includes an interlayer of metal-packaged ceramic tiles and a UHMWPE laminate. We find that:

(1) The composite structure with a metal-packaged ceramic interlayer and UHMWPE laminate effectively resists the impact load of steel projectiles at 592 m/s, and its anti-penetration performance is 50% stronger than that of the traditional composite structure with the same areal density.
(2) The metal-packaged ceramic interlayer absorbs more than one-third of the projectile energy. The metal frame not only provides a sufficiently strong confinement for the ceramic tiles but also limits the growth of ceramic cracks. Therefore, after the penetration, only the ceramic tile which was hit by the projectile in the metal-packaged ceramic interlayer was sufficiently damaged to absorb energy. However, other tiles did not suffer extensive damage, which makes the composite structure maintain high integrity.
(3) Moreover, the UHMWPE laminate absorbs about 39% of the kinetic energy of the projectile through large deformation, which is the highest among all components of the composite structure. By using metal and a UHMWPE composite back plate, it can change the distribution of the impact load in the composite structure, which results in a wider range of circumferential cracks on the top of the fracture cone in the ceramic tile than the target without UHMWPE laminate. Larger circumferential cracks can not only increase the absorbed projectile energy by expanding the crushing range of the ceramic tiles but also prolong the residence time of the projectile on the ceramic surface because it delays the ceramic tiles reaching the tensile limit.
(4) Simulation results reveal that the anti-penetration performance of the composite structure varies with the position of impact, but the difference is no more than 10%. The observed difference is mainly due to the degree of ceramic fragmentation and the deformation of the UHMWPE laminate.
(5) Compared with the metal back plate, the UHMWPE laminate can prevent the fire and ceramic fragments from passing through because the UHMWPE laminate is capable of high elastic recovery ability. In addition, the asymmetrical deformation of the UHMWPE laminate causes notable ballistic deflection. As a result, the UHMWPE laminate can mitigate after-effect damage caused by the projectile penetrating the structure.

## Figures and Tables

**Table 1:** The dimensions of the individual parts of the test and reference targets.

	Areal Density (kg/m^2^)	Section (mm)	Front Plate	Metal-Packaged Ceramic Interlayer	Back Plate	UHMWPE Laminate	Ceramic Tiles
Test Target	45.24	Length	200	200	200	300	50
		Width	200	200	200	300	50
		Depth	3	6	1	7	6
Reference Target	46.53	Length	200	200	200	-	50
		Width	200	200	200	-	50
		Depth	3	6	3	-	6

**Table 2:** Experimental results of projectile impacts on the test and reference target.

Num.	Target	Charge (g)	Impact Coordinates * (mm)	Incident Velocity (m/s)	Residual Velocity (m/s)	Residual Mass (g)
R-1	Reference Target	13.5	(0, 6)	608	379	28.00
R-2		13.5	(23, 3)	612	398	28.32
T-V	Test Target	14.0	(8, 0)	673	168	29.72
T-X		13.5	(−2, 8)	592	0	28.14
T-O		13.5	(−40, 8)	612	339	28.74

* The coordinate system takes the geometric center of the target as the origin and the forward flight of the projectile as the positive direction of the *Z* axis (see Figure 1).

**Table 3:** Deformation of the test and reference structure after projectile impact.

Num.	Impact Coordinates *	Front Plate Perforation Diameter	Back Plate Perforation Diameter	UHMWPE Deformation Diameter	Depth of Bulge on UHMWPE
R-1	(0, 6)	16	24	-	-
R-2	(23, 3)	16	23	-	-
T-V	(10, 0)	14	34	48	35
T-X	(−2, 8)	16	43	34	52
T-O	(−40, 8)	18	20	30	28

* Unit for all data entry is mm. Coordinate defined as in Table 2.

**Table 4:** Parameters of metal materials in the Johnson–Cook model [25].

Parameter	Steel	TC4
Density, ρ (g/cm^3^)	7.85	4.45
Shear modulus, G (GPa)	77	41.9
Static yield strength, A (GPa)	1.54	0.9
Strain hardening coefficient, B (GPa)	0.477	0.845
Strain hardening exponent, n	0.26	0.58
Strain rate coefficient, C	0	0.014
Thermal softening exponent, m	1.0	0.753
Damage constant, D1	2.0	0.05
Damage constant, D2	0	0.27
Damage constant, D3	0	−0.48
Damage constant, D4	0	0.014
Damage constant, D5	0	3.8

**Table 5:** Parameters of ceramic material in the Johnson–Holmquist-2 model [25].

Parameter	SiC	Parameter	SiC
Density, ρ (g/cm^3^)	3.215	Maximum tensile pressure strength, T (GPa)	0.75
Shear modulus, G (GPa)	183	Pressure at the HEL, PHEL (GPa)	14.567
Intact strength coefficient, A	0.96	Damage coefficient, D1	0.48
Fracture strength coefficient, B	0.35	Damage exponent, D2	0.48
Strain rate coefficient, C	0.0045	Bulk modulus, K1 (GPa)	217.2
Fracture strength exponent, M	1.0	Pressure coefficient, K2 (GPa)	0
Intact strength exponent, N	0.65	Pressure coefficient, K3 (GPa)	0

**Table 6:** Parameters of the UHMWPE laminate [38].

Parameter	UHMWPE Laminate
Density, ρ (g/cm^3^)	0.97
Young’s modulus in a-direction, E1 (GPa)	30.7
Young’s modulus in b-direction, E2 (GPa)	30.7
Young’s modulus in c-direction, E3 (GPa)	1.97
Bulk modulus of failed material, Kfail (GPa)	2.2
Longitudinal tensile strength, a-axis, Xt (GPa)	3.0
Transverse tensile strength, b-axis, Yt (GPa)	3.0
Shear strength, ab plane, Sc (GPa)	0.36
Transverse compressive strength, b-axis, Yc (GPa)	2.5
Poisson’s ratio, ba, ν12	0.008
Poisson’s ratio, ca, ν13	0.044
Poisson’s ratio, cb, ν23	0.044
Shear modulus, ab, G12 (GPa)	0.73
Shear modulus, bc, G13 (GPa)	0.67
Shear modulus, ca, G23 (GPa)	0.67
Normal tensile strength, Sn (GPa)	0.95
Transverse shear strength, Syz (GPa)	0.95
Transverse shear strength, Szx (GPa)	0.95

**Table 7:** Comparison of ballistic test results with numerical simulation results.

Section	Incident Velocity (m/s)				Residual Velocity (m/s)			
	T-V	T-X	T-O	R-1	T-V	T-X	T-O	R-1
Experiment	673	592	612	608	168	0	339	379
Simulation	673	592	612	608	165	0	328	367
Error	-	-	-	-	3	-	11	12
Relative error	-	-	-	-	1.7%	-	3.2%	3.1%
**Section**	**Perforation Diameter of Front Plate (mm)**				**Depth of the Bulge of the UHMWPE (mm)**			
	**T-V**	**T-X**	**T-O**	**R-1**	**T-V**	**T-X**	**T-O**	**R-1**
Experiment	14	16	18	16	35	52	28	-
Simulation	12.9	14.9	16.8	16.9	30.6	45.7	23.9	-
Error	1.1	1.1	1.2	0.9	4.4	6.3	4.1	-
Relative error	7.8%	6.9%	6.7%	5.6%	12.6%	12.1%	14.6%	-

**Table 8:** Results of projectile impacts on the test target at different positions.

Num.	Impact Coordinates * (mm)	Residual Velocity (m/s)	Depth of Bulge on UHMWPE (mm)	UHMWPE Perforation Diameter (mm)
PA-1	(0, 0)	0	40	Not penetrated
PA-2	(−54, 0)	44	36	13
PA-3	(54, 0)	0	42	Not penetrated
PB-1	(−27, 0)	118	36	15
PB-2	(27, 0)	55	50	13
PC-1	(−27, −27)	212	29	15
PC-2	(27, 27)	174	38	14

* Coordinate defined as in Table 2. Incident velocity is 650 m/s for all targets.

## Data Availability

The raw/processed data required to reproduce these findings cannot be shared at this time as the data also forms part of an ongoing study.
